# The use of IVF/ICSI and risk of postpartum hemorrhage: A retrospective cohort study of 153,765 women in China

**DOI:** 10.3389/fpubh.2023.1016457

**Published:** 2023-03-21

**Authors:** Di Tang, Yufeng Cheng, Xiaosheng Feng, Xiaocui Li, Peter C. Coyte

**Affiliations:** ^1^College of Public Health, Shanghai University of Medicine & Health Sciences, Shanghai, China; ^2^Shanghai Key Laboratory of Maternal Fetal Medicine, Shanghai First Maternity and Infant Hospital, School of Medicine, Tongji University, Shanghai, China; ^3^Institute of Health Policy, Management and Evaluation, University of Toronto, Toronto, ON, Canada

**Keywords:** assisted reproductive technology, postpartum hemorrhage, retrospective cohort study, pregnant women, *in vitro* fertilization

## Abstract

**Objective:**

Postpartum hemorrhage (PPH) is the leading cause of maternal morbidity and mortality. Identifying women who are at high risk of PPH is crucial for implementing early preventive and interventive strategies. This study aimed to examine whether there is an association between the use of *in vitro* fertilization (IVF) /intracytoplasmic sperm injection (ICSI) and increased risk of PPH.

**Method:**

This retrospective cohort study was conducted using medical record data from women who delivered at a tertiary hospital in Shanghai, China, between January 1, 2013 and April 30, 2019. Logistic regression analysis was used to estimate the associations between the use of IVF/ICSI and the risk of PPH.

**Results:**

A total of 153,765 pregnant women were included, of which 6,484 conceived through IVF/ICSI and147,281 conceived naturally. The incidence of PPH was 1.9% in this cohort. The incidence of PPH in women who conceived through IVF/ICSI was significantly higher than those in women who conceived naturally (3.4% vs. 1.7%, *p* < 0.01). The use of IVF/ICSI was associated with an increase in the amount of postpartum blood loss. Compared to women who conceived naturally, the average amount of postpartum blood loss increased by 42.1 mL (*β* = 42.1, 95% CI, 38.2–46.0) for women who conceived through IVF/ICSI. In addition, women who conceived through IVF/ICSI were at higher risk of maternal PPH. The adjusted odds ratio (OR) of PPH in women who conceived through ART was 2.7 (OR = 2.7, 95% CI, 2.3–3.1).

**Conclusion:**

Our findings demonstrated that women who conceived through IVF/ICSI were at higher risk of PPH and suggested to obstetricians and midwives to identify and implement early preventative strategies for PPH among pregnant women who conceived through IVF/ICSI.

## Introduction

In recent years, extensive evidence has suggested a global upward trend in infertility (1–3). Reproductive-aged couples increased their use of assisted reproductive technology (ART), such as *in-vitro* fertilization (IVF) and intracytoplasmic sperm injection (ICSI), to treat infertility for the past 30 years (4, 5).

With the increasing success rate of ART, more and more attention has been paid to the safety of ART treatment (5). It is well recognized that the use of ART increases the incidence of adverse birth outcomes, including preterm birth (PTB) (6, 7), low birth weight (LBW) (8–10) and some birth defects (11–13). Regarding maternal health outcomes, some recent studies reported that women who underwent ART were at increased risk of pregnancy complications, such as gestational hypertension, preeclampsia and gestational diabetes (GDM) compared with women who conceived spontaneously (14, 15).

Postpartum hemorrhage (PPH) is one of the most common maternal complications of pregnancy. It is the leading cause of maternal morbidity and mortality, and has posed critical health challenges globally (16–18). However, only three studies with different diagnostic criteria of PPH have examined the associations of ART treatment with the risk of maternal PPH, and those three studies all demonstrated that pregnant women who underwent ART were at higher risk of PPH (14, 19, 20). In this study, we examined the associations between the use of IVF/ICSI and risk of maternal PPH among a large cohort of 153,765 Chinese pregnant women.

## Methods

### Study participants

Data in this retrospective cohort study were derived from the electronic medical record system of the Shanghai First Maternity and Infant Hospital (one of the largest prenatal care providers in Shanghai, China, with about 25,000 deliveries per year). The hospital’s electronic medical record system included data on maternal demographic characteristics, reproductive history, pregnancy complications and pregnancy outcomes. We used data of all women who gave birth between January 1, 2013 and April 30, 2019. After excluding multiple deliveries to the same woman, women with stillbirth, undergoing labor induction, delivery with fetal anomalies or women with missing data (*N* = 16,911), 153,765 pregnancies were included in this analysis. This study was approved by the Ethics Committee of the Shanghai First Maternity and Infant Hospital. Individual consent was waived with permission from the ethics committee because all data were deidentified.

### Exposure and outcome definitions

Blood loss was measured gravimetrically after each delivery as follows. Conical drapes were placed at the end of the bed to collect blood lost during and after each vaginal delivery. During cesarean section, a nurse counted the number of blood soaked cotton gauzes and assessed the blood collected in a container. Nurses also measured blood loss during ward rounds. Two specific outcomes were identified: a continuous outcome that measured the volume of postpartum blood loss; and a binary outcome of PPH or not. Specifically, we estimated the effect of the use of IVF/ICSI on those two outcomes: (1) postpartum blood loss (in mL), defined as the amount of postpartum bleeding within 24 h of delivery; (2) PPH, defined as blood loss ≥500 mL for vaginal delivery or blood loss ≥1,000 mL for cesarean section. This definition was based on guidelines from the Chinese Society of Obstetrics and Gynecology and Chinese Medical Association (21).

The use of ART is hereby defined as the use of technology, including *in vitro* treatment of human oocytes or sperm, to assist human reproduction in the treatment of infertility (22, 23). These treatments include *in vitro* fertilization (IVF) and intracytoplasmic sperm injection (ICSI).

### Covariate variables

In all analyses, we adjusted for maternal demographic factors, pregnancy-related complications and reproductive history that have been suggested to be associated with postpartum bleeding (24–27). Those covariate variables included maternal age at delivery, parity, gravidity, hypertensive disorders in pregnancy (HDP), GDM, mode of delivery, prolonged second stage of labor, intra-amniotic infection, gestational age at delivery, newborn birthweight, and placental abnormality (placental abruption, placenta previa, low-lying placenta, placenta accrete and increta).

### Statistical analysis

We used two different approaches to estimate the associations between the use of IVF/ICSI and two specific outcomes. First, we used ordinary least squares (OLS) regression analysis to evaluate the association of IVF/ICSI with the amount of postpartum blood loss (continuous variable), and then we used logistic regression analysis to assess the association of IVF/ICSI with the binary outcome of PPH (blood loss ≥500 mL following a vaginal delivery or blood loss ≥1,000 mL following cesarean section).

We subsequently performed stratified analyses to explore whether the associations between the use of IVF/ICSI and two postpartum bleeding outcomes differed according to mode of delivery (vaginal delivery or cesarean section) or maternal age at delivery (<35 years or ≥ 35 years). As a sensitivity analysis to examine the impact of pregnancy complications, we replicated all models restricted to women without HDP or GDM. In another sensitivity analysis, we replicated all models restricted to women without placental abnormality in order to evaluate the impact of placental abnormality. All estimates were performed using the STATA 14. A 2-sided *p* value less than 0.05 was considered statistically significant.

## Results

### Characteristics of the participants

The overall cohort included 153,765 women, of whom 6,484 (4.22%) conceived through IVF/ICSI. The general characteristics of the participants are summarized in Table 1. The majority of women (82.93%) were aged less than 35 years old. The proportion of women above the age of 35 was 2.4-fold higher among women who conceived through IVF/ICSI than those in women who conceived naturally (38.45% vs. 16.13%, *p* < 0.01). Compared to women who conceived naturally, those who conceived through IVF/ICSI were more likely to be primiparous (92.86% vs. 77.45%, *p* < 0.01), more likely to have GDM (17.98% vs. 12.69%, *p* < 0.01) or HDP (10.76% vs. 4.15%, *p* < 0.01) and more like to have placental abnormality including placenta previa/low-lying (5.00% vs. 3.33%, p < 0.01) and placenta accreta/increta (0.79% vs. 0.30%, p < 0.01).

**Table 1:** Individual characteristics of the participants.

Characteristics	Total population (*N* = 153,765)	Natural conception (*N* = 147,281)	Conceived through IVF/ICSI (*N* = 6,484)	*p*-Value^a^
*Maternal age*				
< 35	127,514 (82.93%)	123,523 (83.87%)	3,991 (61.55%)	<0.001^**^
≥ 35	26,251 (17.07%)	23,758 (16.13%)	2,493 (38.45%)	
*Parity*				
1	120,086 (78.10%)	114,065 (77.45%)	6,021 (92.86%)	<0.001^**^
≥ 2	33,679 (21.90%)	33,216 (22.55%)	463 (7.14%)	
*Gravidity*				
< 4	141,710 (92.16%)	135,797 (92.20%)	5,913 (91.19%)	0.003^**^
≥ 4	12,055 (7.84%)	11,484 (7.80%)	571 (8.81%)	
GDM	19,862 (12.92%)	18,696 (12.69%)	1,166 (17.98%)	<0.001^**^
HDP	6,808 (4.43%)	6,110 (4.15%)	698 (10.76%)	<0.001^**^
*Mode of delivery*				
Vaginal delivery	89,614 (58.28%)	88,064 (59.79%)	1,550 (23.90%)	<0.001^**^
Cesarean section	64,151 (41.72%)	59,217 (40.21%)	4,934 (76.10%)	
Forceps/vacuum delivery	1,802 (1.17%)	1,727 (1.17%)	75 (1.16%)	0.907
*Gestational age*				
Preterm birth	9,159 (5.96%)	7,935 (5.39%)	1,224 (18.88%)	<0.001^**^
Full-term birth	144,606 (94.04%)	139,346 (94.61%)	5,260 (81.12%)	
Placental abruption	35 (0.02%)	34 (0.02%)	1 (0.02%)	0.689
Placenta previa/low-lying	5,222 (3.40%)	4,898 (3.33%)	324 (5.00%)	<0.001^**^
Placenta accreta/increta	496 (0.32%)	445 (0.30%)	51 (0.79%)	<0.001^**^

a *p* values were calculated by chi-square test. ^**^p < 0.01.

### Distributions of the amount of postpartum blood loss or PPH

The amount of postpartum blood loss within 24 h of delivery was measured. As shown in Table 2, the mean amount of postpartum blood loss was 315.2 (155.7) mL. Analyses of potential differences in the amount of postpartum blood loss between the two groups showed that the amount of postpartum blood loss was significantly higher in women who conceived through IVF/ICSI than those in women who conceived naturally (372.6 vs. 312.7, *p* < 0.01).

**Table 2:** Distributions of the amount of postpartum blood loss and PPH.

Outcomes	Total population (*N* = 153,765)	Natural conception (*N* = 147,281)	Conceived through IVF/ICSI (*N* = 6,484)	*p*-Value^a^
Postpartum blood loss (mL)	315.2 ± 155.7	312.7 ± 150.2	372.6 ± 243.4	<0.001^**^
PPH				
Yes	2,990 (1.9%)	2,561 (1.7%)	218 (3.4%)	<0.001^**^
No	150,775 (98.1%)	144,720 (98.3%)	6,266 (96.6%)	

a *p* values were calculated by Mann–Whitney test and chi-square test. ^**^p < 0.01.

According to the diagnostic criteria for maternal PPH, we identified 2,990 cases of PPH and the incidence of PPH was 1.9% in this cohort. The incidence of PPH in women who conceived through IVF/ICSI was significantly higher than those in women who conceived naturally (3.4% vs. 1.7%, *p* < 0.01).

### Associations of IVF/ICSI with the amount of postpartum blood loss or PPH

Table 3 showed the associations between the use of IVF/ICSI and the amount of postpartum blood loss. The use of IVF/ICSI was associated with a significant increase in the amount of postpartum blood loss. Compared to women who conceived naturally, the average amount of postpartum blood loss increased by 42.1 mL (*β* = 42.1, 95% CI, 38.2–46.0) for women who conceived with the use of IVF/ICSI.

**Table 3:** Adjusted associations between the use of IVF/ICSI and the amount of postpartum blood loss or risk of PPH.

	Effect estimates and 95% CI^a^	
	Postpartum blood loss (mL)	Postpartum hemorrhage (PPH)
Natural conception	0 (Reference)	1.00 (Reference)
Conceived through IVF/ICSI	43.8 (39.9–47.7) ^**^	2.73 (2.35–3.18) ^**^

a Models adjusted for maternal age at delivery, parity, gravidity, pregnancy complications, intra-amniotic infection, mode of delivery, prolonged second stage of labor, gestational age at delivery, newborn birth weight and placental abnormality. ^**^p < 0.01.

We subsequently examined the associations between the use of IVF/ICSI and risk of PPH. As shown in Table 3, the use of IVF/ICSI was found to be associated with increased odds of PPH. Compared to women who conceived naturally, the odds of PPH were doubled in women who conceived through IVF/ICSI and the adjusted OR was 2.7 (OR = 2.7, 95% CI, 2.3, 3.1).

### Stratified and sensitive analysis

Table 4 showed the associations of IVF/ICSI with postpartum hemorrhage stratified by mode of delivery (vaginal delivery or cesarean section) or maternal age at delivery (< 35 years or ≥ 35 years). The associations of IVF/ICSI with risk of PPH were generally similar across different modes of delivery or different maternal age (overlapping confidence intervals). While we found maternal age at delivery modified the associations of IVF/ICSI with the amount of postpartum blood loss or PPH, and the observed associations were stronger among women aged less than 35 years old.

**Table 4:** Adjusted associations between the use of IVF/ICSI and the amount of postpartum blood loss or risk of PPH stratified by mode of delivery or maternal age at delivery.

	Effect estimates and 95% CI^c^	
	The amount of postpartum blood loss	PPH
*Mode of delivery**^a^*		
Vaginal delivery	47.7 (41.4–54.0) ^**^	2.8 (2.2–3.4) ^**^
Cesarean section	37.4 (31.9–42.8) ^**^	2.7 (2.1–3.3) ^**^
*Maternal age at delivery**^b^*		
< 35	54.0 (49.4–58.5) ^**^	3.4 (2.9–4.1) ^**^
≥ 35	26.4 (17.8–34.9) ^**^	1.7 (1.2–2.2) ^**^

a Models adjusted for maternal age at delivery, parity, gravidity, pregnancy complications, intra-amniotic infection, prolonged second stage of labor, gestational age at delivery, newborn birth weight and placental abnormality.

b Models adjusted for parity, gravidity, pregnancy complications, intra-amniotic infection, mode of delivery, prolonged second stage of labor, gestational age at delivery, newborn birth weight and placental abnormality.

c Natural conception group was served as reference.

In sensitivity analyses, when restricting all analysis to women without pregnancy complications, the associations of IVF/ICSI with postpartum blood loss or PPH were generally similar with those observed in all participants (Supplementary Table S1). Further, restricting the analysis to women without placental abnormality yielded generally similar results with those observed in all participants (Supplementary Table S2).

## Discussion

PPH, especially severe PPH, contributes substantially to maternal morbidity, causing >50% of all events of severe maternal morbidity (28). Studies have shown an increasing trend in PPH, but the causes for the increase are still uncertain (29, 30). In this large retrospective cohort of 153,765 Chinese pregnant women, we compared PPH between women who conceived through IVF/ICSI and corresponding controls who conceived naturally. We found a significant increase in the amount of postpartum blood loss and higher risk of PPH among women who conceived through IVF/ICSI. Our findings suggest that women who conceived through IVF/ICSI should be allocated to delivery hospitals where adequate preventive and treatment measures are available for PPH.

Globally, an increasing proportion of pregnancies are conceived by ART, and therefore, more concerns are paid on the possible iatrogenic side effects of the treatment (31). We are aware that three previous studies have examined the associations between the use of ART and PPH (14, 19, 20). In one study conducted in Victoria, Australia, Healy DL et al., matched ART clinic data to birth certificate data for single births and found obstetric hemorrhages were more common in women who conceived through ART (20). In another study, Nyfløt Lt et al., used a case–control design of 3,123 women at the Oslo University Hospital and Drammen Hospital in Norway and observed an increased risk of severe PPH in women who conceived through ART procedures (19). Our results were consistent with those of previous studies.

Our stratified analysis indicated greater effects of IVF/ICSI on postpartum hemorrhage in younger women. It seems advanced maternal age protected against postpartum bleeding. However, it is difficult to draw this conclusion based on current evidence. Older women have reduced uterine vascularity, which may decrease PPH risk (32, 33). However, reduced uterine vascularity may also pose an increased risk for thinner endometrium and abnormal placentation, which in turn increases the risk of PPH. Our study suggests that it is imperative to implement intensive preventions and interventions (early cord clamping, controlled cord traction and continuous uterine massage) for younger pregnant women who conceived through IVF/ICSI.

Although the risk of PPH was found to be increased in women who conceived through IVF/ICSI, whether the increased risk was due to IVF/ICSI treatment themselves or maternal factors associated with infertility, was still unclear. In the study by Hayashi M et al., the authors reported that women who conceived through ART procedures were at increased risk for several adverse obstetric and perinatal outcomes, regardless of the type of ART procedure used, and concluded that maternal factors associated with infertility may contribute to the adverse outcomes rather than the ART treatment themselves (34). However, this study only focused on intrauterine insemination (IUI) and IVF and was unable to identify ICSI cycles. While a large proportion of women undergoing ICSI may have no maternal factors related to infertility. In another study by Romundstad LB et al., within unselected population, the authors found that placenta previa occurred 6 times more often in singleton pregnancies after ART compared with naturally conceived pregnancies. Further, by comparing consecutive pregnancies, where the mother conceived spontaneously in one pregnancy and after ART in the other, they still observed a nearly 3-fold higher risk of placenta previa in pregnancy following ART (31). They concluded that the higher risk of adverse pregnancy outcomes is most likely due to a combination of ART treatment and maternal factors associated with infertility.

Previous studies have shown that ART procedures may interfere with the formation of the maternal-fetal interface and disrupt the placentation (31, 34). In our results, placental abnormalities including placenta previa/low-lying (5.0% vs. 3.3%, *p* < 0.01) and placenta accrete/increta (0.79% vs. 0.30%, p < 0.01) are more likely to occur in women who conceived through ART. Those placental abnormalities are known risk factors of PPH (35–37). Additionally, several maternal factors including endometriosis, adenomyosis and hysteromyoma that associated with infertility are also known risk factors of PPH (38–40). Therefore, factors in women requiring ART procedures to conceive may also contribute to the increased risk of PPH. We speculated that the higher risk of PPH among pregnant women who conceived through ART is most likely due to a combination of placental abnormalities and maternal factors related to infertility.

It seems that the difference in blood loss (42 mL) between IVF/ICSI and the natural conception groups is not clinically significant. However, it has significant clinical implications for woman whose postpartum blood loss are close to 500 mL (vaginal delivery) or 1,000 mL (cesarean section), since a very small changes in postpartum blood loss may be enough to pass the threshold of PPH. To our knowledge, this is the largest study to examine the associations of ART with PPH. Given PPH is the leading cause of maternal morbidity and mortality, our findings may have important public health implications. However, there are also some limitations that warrant attention. First, although all participants were from one of the largest prenatal care providers in Shanghai, China, we could not generalize our findings to other countries due to the differences in race and ethnicity, and socio-economic status across different countries. Second, ART and infertility are sensitive issues, most participants did not report the exactly type of ART they used and the underlying infertility diagnosis. Therefore, we could not examine whether the increased risk of PPH among women conceived through ART procedures was due to ART treatment themselves or maternal factors associated with infertility. Third, we were unable to differentiate women with infertility but using other fertility treatments to conceive. These women would inevitably be included in the natural conceived group, which would bias the results toward null. Fourth, our data were derived from the hospital’s electronic medical record. Due to data inaccessibility, we could not adjust for some unmeasured confounders which may lower the certainty of the observed associations. Finally, this study is only an observation and the involvement of ART in any causal pathway of PPH has not been proven. Further studies are needed to explore the underlying mechanisms.

## Conclusion

We found that women who conceived through ART procedures were at increased risk of PPH. Our findings suggest that obstetricians and midwives identify and implement early preventative strategies for PPH among pregnant women who conceived through ART. Decision-makers need to pay more attention to the issue of PPH as the trend will increase if the rate of infertility continues.

## Data availability statement

The original contributions presented in the study are included in the article/Supplementary material, further inquiries can be directed to the corresponding author.

## Ethics statement

The studies involving human participants were reviewed and approved by Shanghai First Maternity and Infant Hospital. Written informed consent for participation was not required for this study in accordance with the national legislation and the institutional requirements.

## Author contributions

XL conceptualized and designed the study, reviewed and revised the initial manuscript. DT carried out the statistical analyses and drafted the initial manuscript. PC provided technical support for statistical analyses. YC and XF helped acquire the data and assisted in the interpretation of results. All authors contributed to the article and approved the submitted version.

## Funding

This study was supported by the Natural Science Foundation of Shanghai (20ZR1444000) and the Shanghai Hospital Development Center, Promotion and optimal management program for diagnosis and treatment of municipal hospitals (SHDC22022230).

## Conflict of interest

The authors declare that the research was conducted in the absence of any commercial or financial relationships that could be construed as a potential conflict of interest.

## Publisher’s note

All claims expressed in this article are solely those of the authors and do not necessarily represent those of their affiliated organizations, or those of the publisher, the editors and the reviewers. Any product that may be evaluated in this article, or claim that may be made by its manufacturer, is not guaranteed or endorsed by the publisher.
