# Supplementary material for: The use of IVF/ICSI and risk of postpartum hemorrhage: A retrospective cohort study of 153,765 women in China
Source: Front Public Health. 2023 Mar 21;11:1016457. doi: 10.3389/fpubh.2023.1016457 (PMC10071037; doi:10.3389/fpubh.2023.1016457)
Supplement: Supplementary file 1 [file Data_Sheet_1.pdf]

Table S1. Adjusted associations between the use of IVF/ICSI and the amount of postpartum blood loss or risk of PPH among women with no pregnancy complications.

	Effect estimates and 95% CI	
	Postpartum blood loss (ml)	Postpartum hemorrhage (PPH)
Natural conception	0 (Reference)	1.00 (Reference)
Conceived by IVF/ICSI	46.4 (42.0-50.8) **	3.0 (2.5-3.5) **

[a] Models adjusted for maternal age at delivery, parity, gravidity, intra-amniotic infection, mode of delivery, prolonged second stage of labor, gestational age at delivery, newborn birth weight and placental abnormality.

** $p < 0.01$.

Table S2. Adjusted associations between the use of IVF/ICSI and the amount of postpartum blood loss or risk of PPH among women with no placental abnormality.

	Effect estimates and 95% CI	
	Postpartum blood loss (ml)	Postpartum hemorrhage (PPH)
Natural conception	0 (Reference)	1.00 (Reference)
Conceived by IVF/ICSI	42.9 (39.5-46.3) **	3.1 (2.6-3.7) **

[a] Models adjusted for maternal age at delivery, parity, gravidity, pregnancy complications, intra-amniotic infection, mode of delivery, prolonged second stage of labor, gestational age at delivery, newborn birth weight.

** $p < 0.01$.
